# Stromal Heterogeneity in the Human Proliferative Endometrium—A Single-Cell RNA Sequencing Study

**DOI:** 10.3390/jpm11060448

**Published:** 2021-05-22

**Authors:** Suzanna Queckbörner, Carolina von Grothusen, Nageswara Rao Boggavarapu, Roy Mathew Francis, Lindsay C. Davies, Kristina Gemzell-Danielsson

**Affiliations:** 1 Department of Women’s and Children’s Health, Division of Obstetrics and Gynecology, Karolinska Institutet, and Karolinska University Hospital, S-171 64 Solna, Sweden; suzanna.queckborner@ki.se (S.Q.); nageswara.boggavarapu@ki.se (N.R.B.); kristina.gemzell@ki.se (K.G.-D.); 2 Department of Medical Biochemistry and Microbiology (IMBIM), Uppsala University, BMC, Husargatan 3, 752 37 Uppsala, Sweden; roy.francis@nbis.se; 3 National Bioinformatics Infrastructure Sweden (NBIS), Department of Cell and Molecular Biology (ICM), Uppsala University, SciLifeLab, 751 24 Uppsala, Sweden; 4 Department of Laboratory Medicine, Karolinska Institutet, S-141 52 Huddinge, Sweden; celltherex@outlook.com

**Keywords:** endometrium, endometrial regeneration, wound healing, mesenchymal stromal cell, endometrial pericyte

## Abstract

The endometrium undergoes regular regeneration and stromal proliferation as part of the normal menstrual cycle. To better understand cellular interactions driving the mechanisms in endometrial regeneration we employed single-cell RNA sequencing. Endometrial biopsies were obtained during the proliferative phase of the menstrual cycle from healthy fertile women and processed to single-cell suspensions which were submitted for sequencing. In addition to known endometrial cell types, bioinformatic analysis revealed multiple stromal populations suggestive of specific stromal niches with the ability to control inflammation and extracellular matrix composition. Ten different stromal cells and two pericyte subsets were identified. Applying different R packages (Seurat, SingleR, Velocyto) we established cell cluster diversity and cell lineage/trajectory, while using external data to validate our findings. By understanding healthy regeneration in the described stromal compartments, we aim to identify points of further investigation and possible targets for novel therapy development for benign gynecological disorders affecting endometrial regeneration and proliferation such as endometriosis and Asherman’s syndrome.

## 1. Introduction

The endometrium is a complex tissue that cyclically regenerates every menstrual cycle in preparation for embryo implantation. Though there is a wealth of research into understanding the endometrial mechanisms involved in the implantation event, far less is known about the tissue’s regenerative properties, akin to scarless wound healing, observed in the proliferative phase. This is important knowledge not only to understand normal endometrial physiology, but also for deciphering the pathophysiology in conditions with impaired endometrial regeneration and proliferation such as Asherman’s syndrome and endometriosis.

Stromal cells are the most abundant cell type in the endometrium and make up the mass of the regenerative endometrial *functionalis*. However, there is limited knowledge of the endometrial stromal compartment in terms of stromal–immune cell interactions, possible niches and functional subtypes. So far only one perivascular stromal cell population has been separately characterized, whereby cell surface marker expression and the transcriptomic profile were described [1,2]. This stromal subpopulation expresses melanoma cell adhesion molecule (MCAM/CD146), platelet derived growth factor receptor beta (PDGFRB) and/or sushi domain containing 2 (SUSD2) [2,3]. It has also been found to exhibit some of the characteristics seen in mesenchymal stromal cells (MSCs) isolated from the perivascular environment of other tissues e.g., adipose tissue and umbilical cord tissue using thymocyte antigen 1 (THY1/CD90), ecto-5′-nucleotidase (CD73) and endoglin (CD105) [2,4]. Frequently, these cells are considered the endometrial stromal progenitor cells [1]. Apart from this population, the majority of the stromal compartment remains widely unexamined.

In a recent study, we sought to characterize endometrial stromal cells (eSCs) in terms of their immunomodulation of T cells and cytokine production in the proliferative phase [5]. In doing so, we found that all expanded eSCs express MSC surface markers thus making the distinction between fibroblasts and progenitor cells based on MSC cell surface markers problematic. eSCs were also shown to be unique in their immune interactions compared to other MSCs, especially in terms of their lack of histocompatibility complex class II (HLA-II) expression post pro-inflammatory stimulation [5]. This supports previous findings describing the endometrium as a unique inflammatory milieu whereby immune cells are carefully regulated throughout the menstrual cycle [6] to promote endometrial repair instead of scarring [7].

In other tissues housing a complex stromal compartment, for example lung [8,9], prostate [10] and lymph node [11] tissue, a diverse number of stromal subtypes with distinguishable features have been identified. Subtypes have been linked to niche and tissue location [11], as well as, specific functional properties such as the response to injury by pericyte subsets [12]. Given this, it is likely that the endometrial stroma holds the same level of complexity and diversity to enable the scarless repair and controlled inflammatory state of the menstrual cycle.

In the present study we set out to further investigate the endometrial stromal compartment in the proliferative phase. Using an unbiased single-cell approach and bioinformatics, we aimed to draw a systematic map of endometrial stromal subsets with signature marker genes for each sub-type and propose functional characteristics.

## 2. Materials and Methods

### 2.1. Healthy Endometrial Donor Material

The study was approved by the regional ethics committee of Karolinska Institutet, Stockholm, Sweden (ethical approval reference numbers DNR: 2015/367-31/4 and 2017/216-31). Written informed consent was obtained from all participating women. Endometrial proliferative stage *functionalis* samples were obtained from healthy volunteers (*n* = 3), aged 24–32 years. All donors had normal menstrual cycles (25–35 days) and were proven fertile (at least one confirmed spontaneous pregnancy). Women were examined for the absence of hormonal disorders, uterine pathologies (e.g., endometriosis, polycystic ovary syndrome and/or previous infertility records), and sexually transmitted diseases (HIV, *Chlamydia trachomatis*-DNA, and *Gonococci*-DNA). None of the women had used hormonal contraception or an intrauterine device for a minimum of 3 months prior to biopsy. Biopsies were collected on cycle day 7, counted from the onset of menses. Biopsies were obtained without cervical dilation or local anesthesia, using a Pipelle aspirator from Cooper Surgical (Trumbull, CT, USA).

### 2.2. Endometrial Single Cell Isolation

Collected endometrial biopsies were stored in MACS^®^ Tissue Storage Solution (Miltenyi Biotec, Lund, Sweden) at 4 °C until further processing. The sample was washed with phosphate-buffered saline (PBS; Sigma-Aldrich, Taufkirchen, Germany) and minced into 2 mm^3^ pieces. The tissue was gently digested in a filter sterilized Dispase II solution (0.5 U/mL; Sigma Aldrich, Taufkirchen, Germany) in complete media composed of DMEM-F12 (Thermo Fisher Scientific, Dreieich, Germany) with 10% (*v*/*v*) fetal calf serum (FCS; Thermo Fisher Scientific) at 4 °C overnight. The tissue solution was manually disaggregated, washed with complete media and centrifuged at 200× *g* for five minutes. The tissue was further digested with filter sterilized Collagenase III (150 U/mL; Worthington, Lakewood, USA) and DNAse (139 U/mL; Sigma Aldrich) in complete media, with agitation, for 45 min at 37 °C. Once the tissue was completely dissociated, the cells were washed in complete media and centrifuged at 200× *g* for five minutes. The cell pellet was treated with 1 ml of Red Blood Cell Lysis Buffer (Roche, Solna, Sweden) for five minutes at room temperature. The reaction was stopped with the addition of complete media, and the cells washed and centrifuged at 200× *g* for five minutes. Cells were counted and the viability was assessed using the TC20™Automated Cell Counter (BioRad, Gothenburg, Sweden). The cells were resuspended in PBS supplemented with bovine serum albumin (400 µg/mL; Sigma Aldrich) at a cell concentration of 1000 cells/µL.

### 2.3. Single-Cell Library Preparation and mRNA Sequencing (scRNA-Seq) of Proliferative Phase Endometrial Cells with 10× Genomics

The endometrial cell suspensions were delivered to the Eukaryotic Single Cell Genomics Facility (ESCG, SciLifeLab, Stockholm, Sweden), prepared and loaded on a 10× Genomics Chromium Controller instrument for single-cell gel bead-in-emulsion (GEM) formation and barcoding using the Chromium Single Cell 3′ Gel Bead Kit v2. GEM reverse transcription was performed. Once cDNA was generated, amplified by polymerase chain reaction (PCR) and cleaned, the sequencing libraries were constructed according to the manufacturer’s instructions using the Chromium Single Cell 3′ Library Kit v2 (10× Genomics).

Three runs of scRNA-seq of uncultured endometrial cells were performed with one run per sample (*n* = 3). Each run consisted of one sample/sequencing lane using the Illumina 2500 instrument. Approximately 3000 cells were sequenced per sample with an average sequencing depth of 50,000 reads per sample.

### 2.4. ScRNA-Seq Data Analysis

Cell Ranger 2.1.1 was used to process raw sequencing data. Reads were aligned to the hg19 transcriptome using STAR mapper [13] and quantified to counts. Analysis of count data was performed in R 3.6.0 and 3.6.1 using Seurat 3.1.2 and 3.1.3 [14,15]. SoupX [16] was run on the raw counts for each sample separately to estimate levels of background RNA contamination. Initial quality control measures were performed to exclude potential doublets and dying cells. Cells expressing 200–5000 genes, and less than 10% of mitochondrial genes were kept, resulting in 6348 cells in total. Ribosomal genes (RPL and RPS genes) were removed. Cell cycle phase was determined using Seurat. Counts were normalized and the effect of cell cycle genes were regressed out using sctransform [17] within each run. The three runs were integrated together with CCA in Seurat [15] to remove the effect of the batch and the individual. The dimensionality of the data was reduced using principal component analysis (PCA). Elbow plot was used to select the top PCs, which were used downstream for Louvain clustering and visualization using t-distributed stochastic neighbor embedding (tSNE) and uniform manifold approximation and projection (UMAP). The “Reference-Based Single-Cell RNA-Seq Annotation” tool SingleR [18] was run using HPCA (Human Primary Cell Atlas) and BPE (Blueprint ENCODE) reference data to broadly identify cell types by machine learning, thereby avoiding initial labelling bias. Final cell type labels were established after manual assessment using known marker genes from within the endometrial field and wider stromal/vascular literature. Once cells were identified, the marker genes for each subset were determined by differential gene expression tests using Model-based Analysis of Single-Cell Transcriptomics test (MAST) [19]. Larger groups of cells were subset further by downstream analysis using the same Seurat steps. Velocyto was used to evaluate cell lineage by dynamics and RNA velocity [20].

Differences in gene expression between clusters (cell types or subtypes) were statistically quantified through differential gene expression analyses. The MAST test [19] was run in Seurat to identify differentially expressed genes (DEGs) between clusters of interest. A log fold change of 2 or 1.5 and a Bonferroni-adjusted *p*-value of 0.05 was applied to determine DEGs.

## 3. Results

### 3.1. Unbiased Single-Cell Analysis Confirms Known Endometrial Cell Types

**Figure 1:**
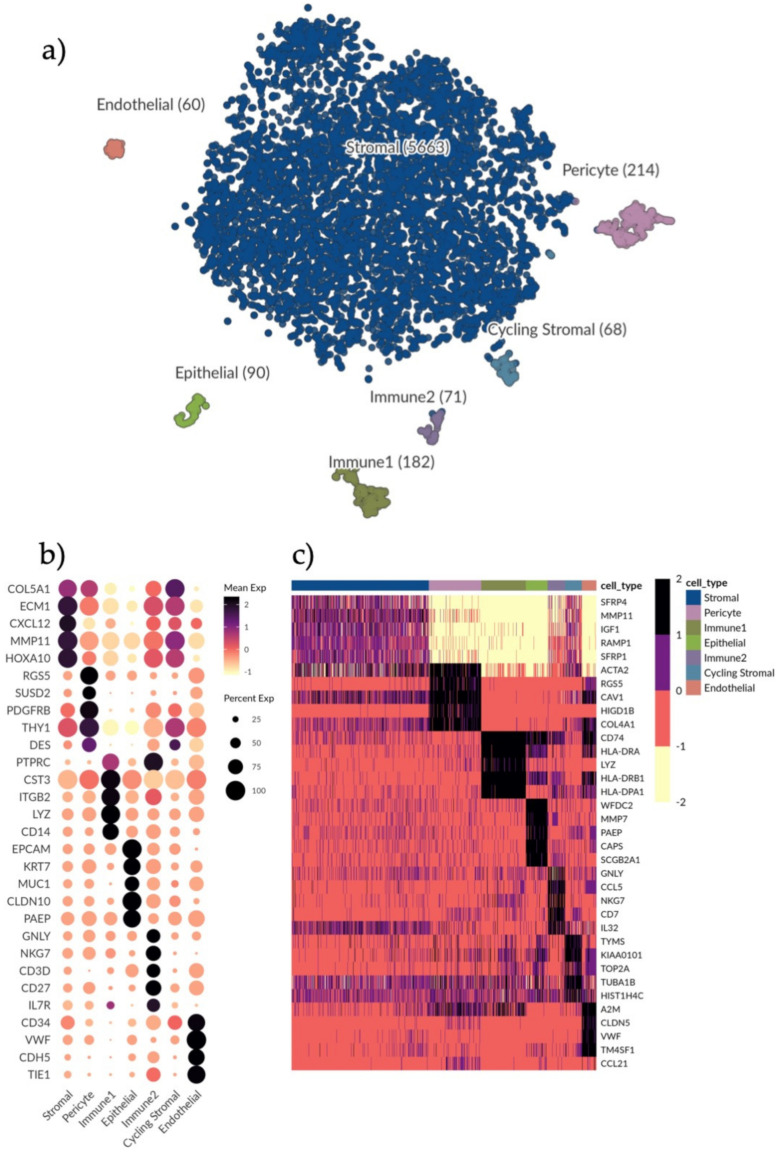
Single-cell clustering and identification of endometrial cells. (**a**) t-SNE plot visualizing clustering of scRNA-seq results from 6348 endometrial cells. Seven clusters were detected, namely, epithelial cells (*n* = 90), endothelial cells (*n* = 60), pericytes (*n* = 214), immune1 (*n* = 182), immune2 (*n* = 71), stromal cells (*n* = 5663) and cycling stromal cells (*n* = 68). (**b**) Dotplot showing the gene expression of additional selected marker genes for each cell type to further identify clusters. Dots denote mean normalized expression values over clusters. Immune1 displays a high expression of genes indicative of a monocyte phenotype, for example *CD14* and *LYZ.* Immune2 displays an expression of *CD27* indicating a T cell/NK cell phenotype. (**c**) Heatmap displaying the top differentially expressed genes (rows) for each cell type (columns) based on the MAST test with a minimum log fold change of 2 and adjusted *p*-value of 0.05. Color scale is clipped at 2.5.

The principal aim of this study was to explore the endometrial stromal compartment, during the proliferative phase (cycle day 7) of the menstrual cycle, to identify stromal cell complexity and diversity. Endometrial biopsies from healthy fertile women (*n* = 3) were collected during the proliferative phase and processed to obtain a single cell suspension (see Materials and Methods). After viability and quality control, isolated cells were submitted for single-cell RNA sequencing (scRNA-seq) on the 10× Genomics Chromium platform (Figure S1a). In order to obtain an unbiased profile of the stromal compartment, cells were not in vitro expanded nor sorted for known stromal and progenitor markers. Background RNA contamination estimates for the three samples were found to be low (1%, 1% and 3%, respectively). Transcriptomic profiles for 6864 cells (2322, 1747 and 2795 per sample) were acquired and were reduced to 6348 (2159, 1610 and 2579 per sample) after further quality control and filtering (see Materials and Methods). Using tSNE to visualize the cells, seven clusters could be isolated with unique transcriptional profiles (Figure 1a). All donors contributed equally to the cell clusters (Figure S1b) while batch effect was corrected for.

In order to identify cell types, we explored the expression of different well-known marker genes in our seven clusters (Figure 1b and Figure S1c). We found unique identifier genes for each cell type: *VWF* identified endothelial cells, *EPCAM* distinguished epithelial cells and *RGS5* identified pericytes. Two clusters were identified as immune cells by expression of *PTPRC/CD45* (Immune1 and Immune2). The main cluster making up the bulk of the sequenced cells was identified as stromal cells with a broad expression of *CXCL12* (Figure S1c). This was to be expected as the tissue digestion protocol was optimized for eSC enrichment and eSCs make up the bulk component of the in vivo endometrial *functionalis* tissue composition. A more extensive characterization of each subset followed with endometrial stromal progenitor and MSC markers (e.g., *SUSD2, PDGFRB, THY1*) most highly expressed in the pericyte subset (Figure 1b). Immune1 was predominantly composed of monocytes (*CD14*) and macrophages, while Immune2 included natural killer (NK)-cells and T-cells (*CD27*) respectively (Figure 1b). Certain cell types, such as B-cells, that we would expect to find in the tissue in vivo could not be identified, which is likely due to their vulnerability to the cell isolation protocol.

### 3.2. Further Characterization of Endometrial Cell Types and Cell-Type Specific Marker Genes

We applied MAST (see Materials and Methods) with a minimum log fold change of two and an adjusted *p*-value of 0.05 to determine the top differentially expressed genes for all identified cell types (Figure 1c). The stromal compartment had the least number of differentially expressed genes relative to the other subsets, suggesting a more diverse composition. Stromal signature genes were: *SFRP1* and *SFRP4* involved in the Wnt-bone morphogenic protein (BMP) signaling pathway; *IGF1*, a growth factor associated with immunomodulation and regeneration; and extracellular matrix (ECM) genes e.g., *MMP11*.

Pericyte signature genes were: *ACTA2*, a marker for smooth muscle actin highly expressed in the vasculature and in fibroblasts; *RGS5*, a known perivascular maker; and *CAV1*, an inhibitor of the TGFβ1 pathway regulating inflammation and ECM production. In line with this, *COL4A1*, which encodes the alpha chain of type IV collagen and is a key player in angiogenesis [21], was also highly expressed in the pericyte cluster.

In Immune1 (monocytes and macrophages) there was a high expression of HLA-II related genes *CD74*, *HLA-DRA*, *HLA-DR1A* and *HLA-DR1B* and bacteriolytic *LYZ* while in Immune2 (T cells and NK cells) genes relating to T cell and NK cell recruitment and activation were highly expressed (*GNLY*, *CCL5*, *NKG7*, *IL32* and *CD7*).

In the epithelial subset, previously identified markers of epithelial wound repair and immunomodulation (*WFDC2*, *PAEP*, *MMP7*) were highly expressed as well as *CAPS* which is involved in cell-signaling, and *SCGB2A1*, an androgen-regulated gene.

In the endothelial subset, endothelial marker genes (*CLDN5* and *VWF*) were highly expressed along with *TM4SF1*, a marker previously associated with activated endothelial cells [22].

A smaller stromal cluster shared the same gene expression profile as the main stromal subset but had higher levels of cell cycle related genes (*TYMS, KIAA0101*, *TOP2A*) as its only distinguishing feature and was thus labelled as cycling stromal cells.

### 3.3. Heterogeneity in the Endometrial Stromal Compartment and Possible Stromal Subtypes Revealed by Single-Cell Analysis

**Figure 2:**
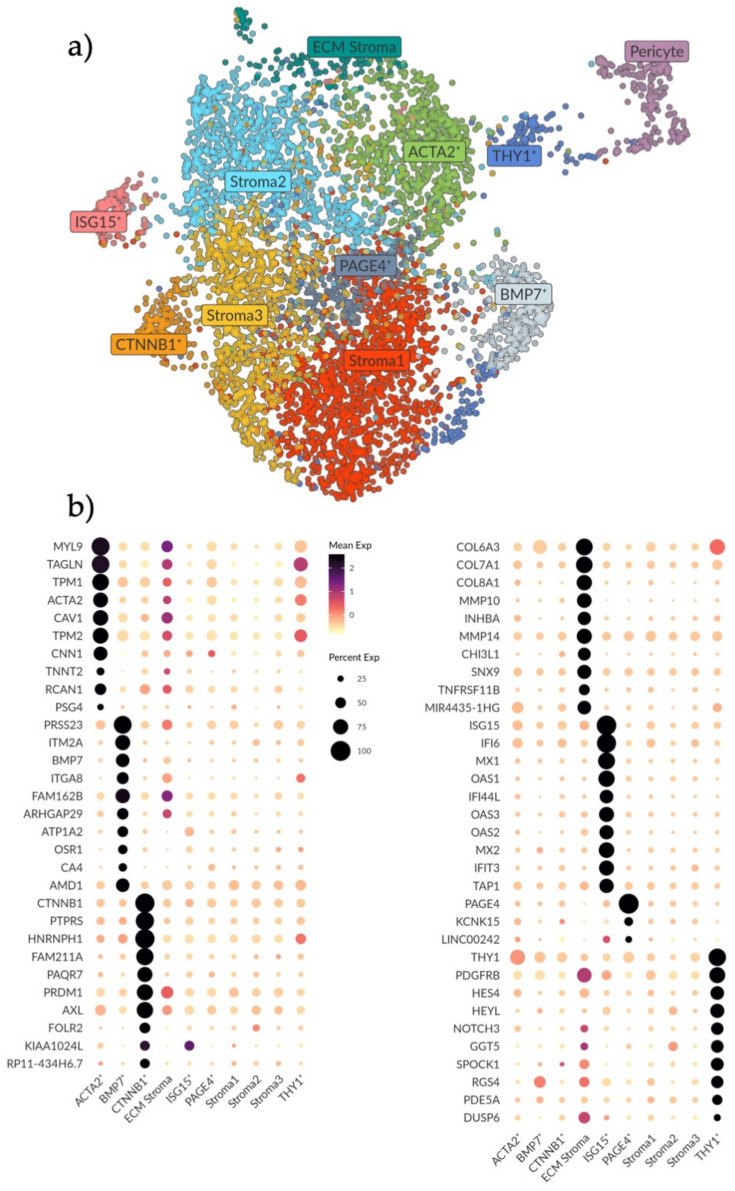
Analysis and subtyping of the endometrial stromal cell compartment. (**a**) UMAP plot showing ten clusters of endometrial stromal cells and a cluster of pericytes on the far right. Clusters are labelled as per their identified expression profile in b. (**b**) Dotplot showing the top differentially expressed genes (rows) for the ten stromal clusters (columns). Genes were selected based on the MAST test with a minimum log fold change of 2 and an adjusted *p*-value of 0.05. Note that the color scale is clipped at 2.5. Stroma 1, 2 and 3 do not show any unique expression. The PAGE4+ cluster shows a very biased differential expression for *PAGE4* only. The ECM, ACTA2+ and BMP7+ clusters show a higher expression for genes involved in the ECM breakdown, remodeling and organization. The CTNNB1+ cluster shows higher expression of genes involved in epithelial regulation and innate immunity. The ISG15+ cluster shows higher expression for genes involved in innate immunity. The THY1+ cluster shows higher expression of genes involved in Notch signaling.

To further characterize the stromal compartment, stromal cells were separated from the main dataset for additional in-depth analysis. As the cycling stromal cells had a different cell cycle phenotype they were excluded from downstream analysis. UMAP and Louvain clustering was rerun to create ten independent populations (Figure 2a). UMAP analysis uses spatial placement to illustrate the association of different clusters with one another. Although perivascular cells are not considered stromal cells *per se*, stromal and perivascular cooperation in regeneration is well established [23] and as such pericytes were included in the initial clustering to determine which stromal clusters they relate to and how (Figure 2a).

Using the MAST test with a minimum log fold change of 2 and adjusted *p*-value of 0.05, the top ten distinguishing genes for each stromal subset were determined to generate a profile (Figure 2b). Three clusters (Stroma 1, 2 and 3) did not exhibit any unique expression profiles compared to the other clusters. We interpret this as these cells being the baseline stromal cells that comprise the bulk of endometrial stroma. Closely related to these clusters we saw one small cluster with a biased expression for *PAGE4* (Figure 2b).

Gene profiles of the remaining six stromal populations were characterized and labelled based on significant genes (Figure 2b). The ACTA2+ population exhibited a gene signature (*MYL9*, *TAGLN, TPM1*, *ACTA2*, *TPM2*, *TNNT2* and *CNN1*) suggestive of activated fibroblasts and smooth muscle cells [24,25], specifically the signature was made up of genes that modulate actin and myosin interactions. Adjacent to this cluster, the ECM population had the highest expression of collagens and matrix metalloproteinases (MMPs) genes (*COL6A3*, *COL7A1*, *COL8A1*, *MMP10* and *MMP14*), which are components of the basement membrane (e.g., surrounding vasculature) and vital in the regulation of tissue remodeling and homeostasis. The BMP7+ population exhibited a signature of genes involved in myofibroblast differentiation, epithelial mesenchymal transition (EMT) and TGFβ1-WNT signaling (*PRSS23*, *ITGA8*, *BMP7*, *ITM2A* and *ARHGAP29*) [26,27,28,29]. We hypothesize that these three stromal clusters (ACTA2+, ECM and BMP7+) represent stromal subtypes that are active in ECM breakdown, remodeling and organization which are important processes during the proliferation, tissue repair and regeneration that occurs in the endometrium during and after menstruation.

In one stromal cluster we noted a high expression of *CTNNB1* (Figure 2b). This gene has been previously linked to Wnt signaling and stromal cell regulation of epithelial proliferation and differentiation in wound healing [30,31]. Other genes in this population have also been associated with epithelial–stromal interactions, as well as innate immune responses, specifically M2 anti-inflammatory macrophage polarization (*PTPRS*, *PAQRF*, *PRDM1*, *AXL* and *FOLR2*) [32,33,34,35]. Similarly, in the ISG15+ population, most genes are involved in interferon signaling and innate immunity functions (*ISG15*, *IF16*, *MX1*, *OAS1*, *IF144L*, *OAS3*, *OAS2, MX2, IFIT3*) [36,37], suggestive of an activated stromal cell state/a subtype active in immunomodulation.

Lastly, one stromal population was distinguished by high *THY1* expression, which is a surface glycoprotein commonly used as an MSC marker (also known as CD90). This cluster also had the highest gene expression associated with Notch signaling (*HES4, HEYL, NOTCH3*), a pathway involved in cell–cell signaling, cell plasticity and differentiation, as well as genes associated with the pericyte (*PDGFRB, PDE5A*) [38,39]. This population was also the one most adjacent to the pericyte cluster and we therefore hypothesize a perivascular location for this population.

### 3.4. Single-Cell Analysis of Endometrial Pericyte Cells Reveals Two Distinct Subtypes

**Figure 3:**
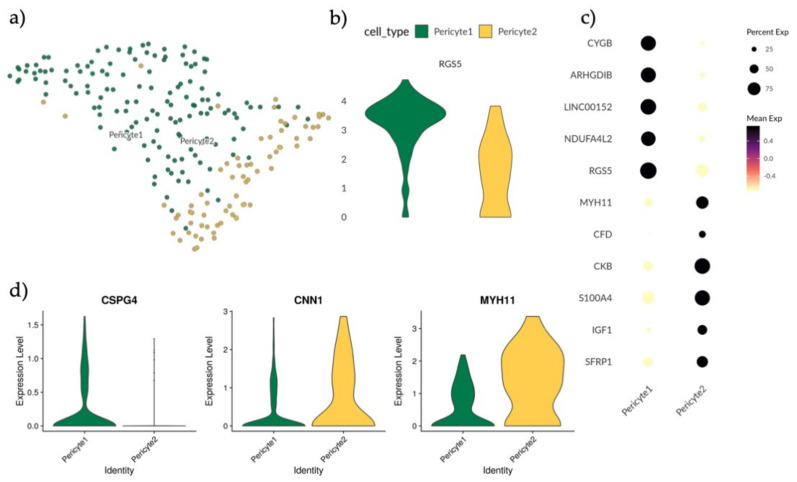
Analysis and subtyping of endometrial pericytes. (**a**) UMAP plot of scRNA-seq results visualizing the clustering of endometrial pericytes into two separate clusters, Pericyte1 (green) and Pericyte2 (yellow). (**b**) Violin plot displaying full distribution of *RGS5* expression in Pericyte1 and Pericyte2. (**c**) Dotplot showing mean expression of top differentially expressed genes for Pericyte1 and Pericyte2 after MAST test with minimum log fold change of 1.5 and an adjusted *p*-value of 0.05. (**d**) Violin plots showing full distribution of marker genes *CSG4, CNN1* and *MYH11* in Pericyte1 and Pericyte2 with the profile *CSPG4^+^ CNN1^low^ MYH11l^ow^* for Pericyte1 and *CSPG^−^ CNN1^high^ MYH11^high^* for Pericyte2.

Using the known pericyte marker *RGS5* we identified one cell population containing pericytes (Figure S2a) [39]. As pericytes have previously been linked to endometrial stromal regeneration we wanted to further explore this cell subset [2,40]. Pericytes were isolated from the main dataset and reanalyzed applying UMAP and Louvain clustering. Along with differential gene expression, we identified two separate populations (Pericyte1 and Pericyte2) (Figure 3a).

In order to explore the identities of Pericyte1 and Pericyte2, the expression levels of the perivascular marker *RGS5* were compared, revealing that Pericyte1 had a higher mean expression than Pericyte2 (Figure 3b). By applying MAST analysis with a minimum log fold change of 1.5 and an adjusted *p*-value of 0.05, the differentially regulated genes between the two populations were determined, with *CYGB, ARGHDIB, LINC00152* and *NDUFA4L2* specific to Pericyte1 and *MYH11, CKB, CFD, S100A4, IGF1* and *SFRP1* specific to Pericyte2 (Figure 3c). MYH11 has previously been used as a marker for mature smooth muscle cells (SMCs) in the perivascular niche [41]. To further investigate if Pericyte2 could represent an SMC-like pericyte subtype, the expression of additional reported markers *CSPG4, CNN1* and *MYH11* in both Pericyte1 and Pericyte2 was validated [41] (Figure 3d). Pericyte1 presented a *CSPG4*^+^ *CNN1*^low^ *MYH11*^low^ profile and Pericyte2 had a *CSPG*^−^ *CNN1*^high^ *MYH11*^high^ profile. This is consistent with Pericyte1 representing a classic pericyte/mural cell population, and Pericyte2 representing an SMC-like or contractile pericyte [41].

### 3.5. Transcriptional Expression of PDGFRB, MCAM, SUSD2 and THY1 Extends across the Greater Perivascular Niche 

**Figure 4:**
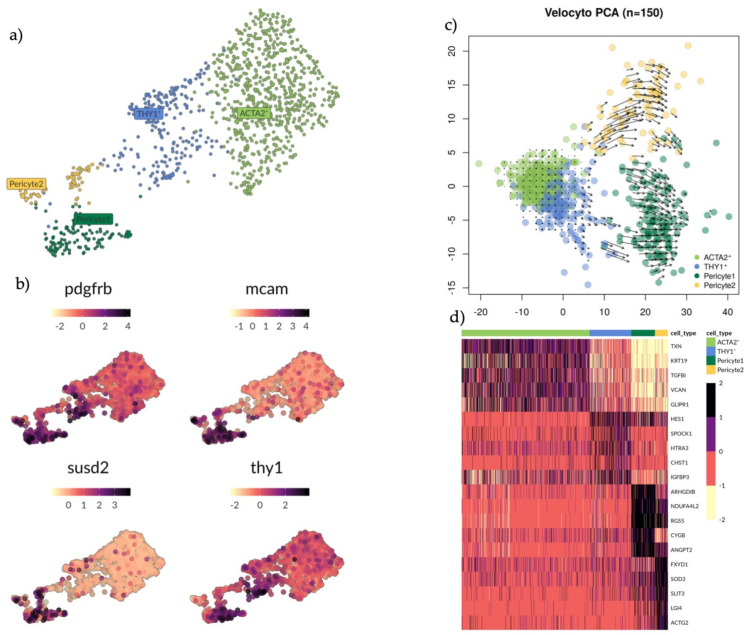
Analysis of subsets ACTA2+, THY1+, Pericyte1 and Pericyte2 clusters with marker exploration and RNA velocity analysis. (**a**) UMAP plot showing groups ACTA2+ (light green), THY1+ (blue) and Pericyte 1 (dark green) and Pericyte 2 (yellow). (**b**) UMAP plot showing expression pattern of genes *PDGFRB, MCAM, SUSD2* and *THY1.* None of these markers can exclusively identify any of the clusters mentioned in A. (**c**) PCA scatterplot showing RNA velocity. Predicted developmental trajectory between clusters is displayed as a vector field. Short arrows indicate a steady state and long arrows indicate active progression towards a differentiated state. Cells differentiate along the direction of the arrow, here it indicates some of the THY1+ cells committing towards the Pericyte1. (**d**) Heatmap showing the top differentially expressed genes (rows) for each cell cluster (columns) based on a MAST test with a minimum log fold change of 1.5 and an adjusted *p*-value of 0.05.

Endometrial stromal regeneration has been said to be orchestrated by stromal progenitor cells in the perivascular environment [42]. As such, we attempted to establish a hierarchy between cell populations (ACTA2+, THY1+, Pericyte1 and Pericyte2) presenting gene profiles indicative of a perivascular and smooth muscle actin identity, thereby establishing more specific marker genes (Figure S2b). *THY1* expression was seen to increase in the stromal subsets with increased proximity to the pericyte as per UMAP analysis, where local and global structures are preserved and thus the distance between the subsets indicates similarity (Figure S2c). The four populations were divided and integrated, UMAP analysis was performed (Figure 4a) and then relative scaled expression across the subset of *PDGFRB, MCAM, SUSD2* and *THY1* was determined (Figure 4b).

*PDGFRB* was highly expressed in Pericyte1 and THY1+ cells. *MCAM* and *SUSD2* were highly expressed in Pericyte1 and Pericyte 2. *THY1* was highly expressed in Pericyte1 and THY1+ cells. No specific marker distinguished the THY1+ cells from other cell populations in the subset (Figure 4b). We applied RNA velocity (Velocyto, see Materials and Methods) analysis on the subset to determine the developmental trajectory (Figure 4c). Within each cell on the PCA plot, the amplitude and direction of the arrow provides information about the developmental trajectory. Pericyte1 and Pericyte2 showed long active arrows with opposing directional progression to different states. Within the THY1+ cells there were two sub-populations: one closer to the ACTA2+ cells, with short arrows suggesting a steady state, while the other subset had longer arrows committing towards Pericyte1. The ACTA2+ cells presented a uniform identity with the shortest arrows overall, possibly an example of a more committed state. Their trajectory was in the opposite direction of Pericyte1 and Pericyte2. Finally, we sought to identify more specific marker genes for the different populations within the perivascular environment so they could be more easily distinguished relative to the other populations in this environment by applying a MAST analysis with a minimum log fold change of 1.5 and an adjusted *p*-value of 0.05 (Figure 4d). *TXN, KRT19, TGFBI, VCAN* and *GLIPR1* specifically identified the ACTA2+ cells. *HES1*, *SPOCK1, HTRA3, CHST1* and *IGFBP3* identified THY1+ cells. *ARHGDIB, NDUFA4L2, RGS5, CYGB* and *ANGPT2* identified Pericyte1. *FXUD1, SOD3, SLIT3, LG14* and *ACTG2* identified Pericyte2. Overall, these gene profiles provide more specific marker genes within the perivascular environment as current gene profiles appear to be more telling of cell location rather than cell type.

### 3.6. Validation of Endometrial Stromal and Pericyte Subtypes in External Data Sets Reveals Distinguishable Subtypes Persisting throughout the Menstrual Cycle and in Early Pregnancy

In order to validate our findings of endometrial stromal and pericyte sub-types we imported external human scRNA-seq data from the fetal-maternal interface (FMI) by Vento-Tormo et al. [43].

**Figure 5:**
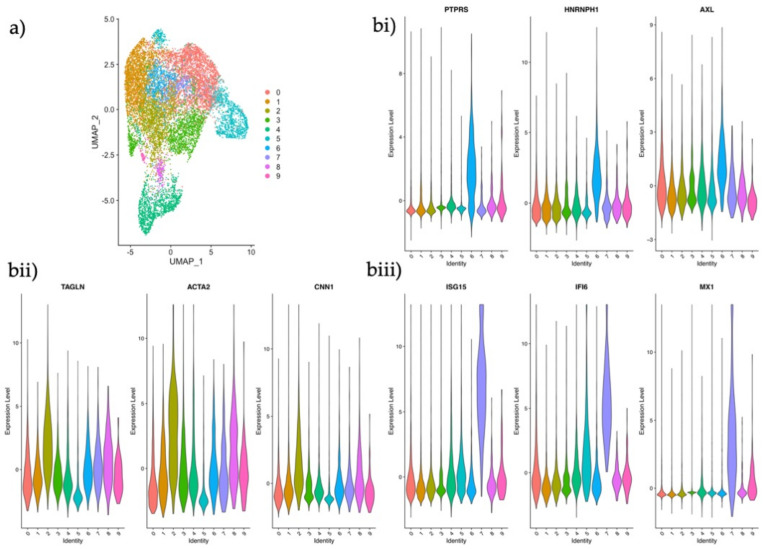
Analysis and identification of endometrial stromal subtypes in the maternal-fetal interface. (**a**) UMAP plot of scRNA-seq data of the maternal *CD45−* decidual fraction from the maternal-fetal interface displaying ten clusters using the same parameters applied in our dataset. (**b**) Violin plots of log2 normalized counts showing marker gene expression for each population. (**i**) CTNNB1+ epithelial regulator; *PTPRS, HNRNPH1* and *AXL* (**ii**) ACTA2+ activated fibroblast; *TAGNLN, ACTA2* and *CNN1* (**iii**) ISG15+ innate immunity stromal subtype; *ISG15*, *IFI6* and *MX1*.

The FMI dataset was filtered for the maternal *CD45-* decidual fraction (12,544 cells) providing a suitable comparison to the endometrial stromal compartment and perivascular cells. Maternal decidua is endometrium following decidualization and placentation. As part of the original analysis three decidual stromal populations had been explored (dS1, dS2 and dS3) and two perivascular populations (dP1 and dP2) (Figure S3). A similar workflow, as initially described, was applied to the subset data of interest. tSNE clustering was used in Seurat to identify 10 clusters (Figure 5a).

In order to investigate if any of these clusters represented the subtypes that had been identified in our data set, we searched for the expression of some of our distinguishing stromal marker genes (Figure 2b) in the maternal decidua clusters. Gene expression profiles identified one cluster as the CTNNB1+ population (Figure 5bi), another as the ACTA2+ population (Figure 5bii), and a third as the ISG15+ stromal subtype (Figure 5biii). These data indicate that some stromal phenotypes may be persistent players in the endometrial stromal compartment homeostasis and in early pregnancy.

**Figure 6:**
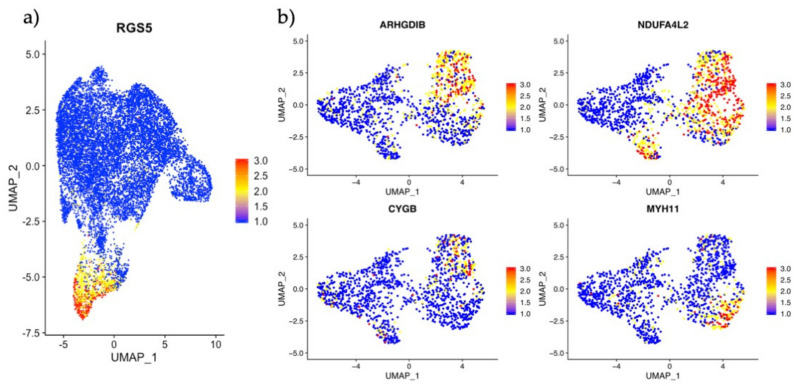
Analysis and identification of endometrial pericyte subtypes in the maternal-fetal interface. (**a**) Scatter plot showing *RGS5* expressing cells in the UMAP projection. (**b**) Scatter plot of selected *RGS5+* cells and their scaled gene expression of *CYGB, ARGHDIB, NDUFA4L2* and *MYH11* identifying the top right cells as Pericyte1 and the bottom right cells as Pericyte2.

In order to investigate our endometrial pericyte subtypes in early pregnancy, cells were selected for *RGS5* expression in the external dataset (Figure 6a). Using the markers *CYGB, ARGHDIB* and *NDUFA4L2* we could readily identify one cell cluster as corresponding to Pericyte1 and by using the *MYH11* expression we could identify Perictye2 (Figure 6b).

## 4. Discussion

Understanding healing and growth mechanisms is crucial when explaining deregulation in benign gynecological disorders which present an inflammation/proliferation imbalance [44,45]. Using unbiased single-cell transcriptional data, this study demonstrates that the nature of the endometrial stromal compartment is more complex than previously assumed. Undeniably, the endometrial perivascular environment holds many unanswered questions about its role as a progenitor cell niche and regulator of endometrial regeneration. However, equally important are the other niches which coordinate controlled stromal proliferation, the ECM’s ever-changing composition and inflammatory homeostasis. The gene expression signatures of the stromal subsets we have described in this work; of which three are retained throughout decidualization and early pregnancy, provide a starting point towards understanding the complexity of the greater stromal compartment. At this point, further follow-up experiments are required to establish whether transcriptomic heterogeneity in a cell population is indicative of previously unknown cell types, cell states or cell niches in vivo. All our stromal subsets warrant translational validation and further exploration to provide functional meaning to the transcriptional profiles.

Previously, we observed that eSCs have a unique immunomodulatory phenotype, characterized by a lack of HLA class II cell surface expression in pro-inflammatory conditions (IFNγ and TNF-α licensing) in contrast to other stromal progenitor cells [46,47,48]. Furthermore, they limit inflammation by reducing CD4+ T helper cell proliferation while shifting them towards an effector memory phenotype [5]. In the current study, the ISG15+ population, in particular, revealed an interferon regulated gene expression profile suggestive of an activated stromal population, as seen in inflammatory settings such as tissue regeneration following menstruation [49]. Similarly, the CTNNB1+ population included genes linked to M2 macrophage polarization e.g., *AXL, FOLR2* [33,34,35]. M2 macrophages are involved in remodeling tissue and secreting anti-inflammatory cytokines, with evidence suggesting stromal cells may be able to polarize macrophages to an M2 state via paracrine mechanisms [50,51]. Immunomodulation studies in vitro could provide us with a better understanding of the ISG15+ population and CTNNB1+ populations. Additionally, computational tools like CellPhone DB, with extensive endometrial immune cell sequencing data, could provide further information about specific eSC mechanisms in tissue regeneration and the immunomodulation of specific lymphocytes [52].

The ACTA2+ subset clearly distinguished itself from other stromal subsets by its high expression of smooth muscle actin and myosin regulating genes. Further delineation of the population’s identity was complicated by the inability to discriminate between a perivascular smooth muscle location, an activated fibroblast or a terminally differentiated myofibroblast based on a transcriptional profile alone. ACTA2 has historically been used as a marker in multiple settings [24,25]. Based on the RNA-velocity analysis, ACTA2+ cells presented a steady state with the lowest differentiation capacity relative to other cell populations in the perivascular environment, aligning their identity more with a myofibroblast. ACTA2+ cells were also highlighted in the analysis by Vento-Tormo et al. with gene and protein expression observed in the perivascular environment, as well as, the decidua spongiosa adjacent to the myometrium [43]. Thus, there is a general need to find specific markers to discriminate between smooth-muscle proximity and myofibroblast activation to better determine each of these cells’ role in regeneration [53].

Within the endometrial pericyte we found two subsets, with Pericyte1 potentially representing a more classical pericyte/mural cell while Pericyte2 revealed a gene profile more aligned with vascular SMCs as per the gene profiles introduced by Kumar et al. [41]. However, the greater pericyte field has no specific marker profile which indisputably, and regardless of developmental stage, identifies pericytes from vascular SMCs or MSCs [53,54]. Common markers include ACTA2, PGDFRB, DES, RGS5 and CSPG4 [53]. This leads to the question of where the perivascular cells end and the stromal compartment with myofibroblasts and progenitors begins. PDGFRβ, MCAM [2] and SUSD2 [4] have previously been used as markers in the endometrium to identify stromal progenitors, however our data suggests that on a gene level these markers are expressed by all cells in the perivascular environment, with the highest gene expression in the Pericyte1 and Pericyte2 populations. This was similarly observed in the extensive single cell sequencing study of the whole menstrual cycle recently published by Wang et al. [55]. Further investigation as to whether these markers are more discriminatory at a protein level is needed, especially as stromal progenitors and pericytes are isolated and enriched in vitro using similar cell-surface markers. THY1/CD90 in combination with CD73 and CD105 are used as MSC markers to identify endometrial stromal progenitors [56]. Although our data shows differential expression of *THY1* across the stromal compartment and pericyte, it is not exclusive to a progenitor cell or the pericyte. THY1 is expressed on the cell surface of a number of cells, including fibroblasts [5,57], and while it is associated with undifferentiated states in stromal and hematopoietic cells, it is a marker for progressive maturation in neurons [58]. This demonstrates the complexity of its biological role beyond being a marker and the importance of the cellular microenvironment [58]. Overall, our findings suggest that existing endometrial markers for progenitor cells in the perivascular environment are not specific enough on a gene level to distinguish between key cells in this niche. This makes it difficult to determine their individual role in regeneration.

A possible reason why multiple stromal populations could not be validated in the external dataset was due to the considerable effect of decidualization on stromal cell morphology and their immunophenotyped. Equally, the tissue dissociation process and the choice of digestive enzymes has previously been shown to alter the transcriptome, this may lead to the differences seen between endometrial datasets. Bioinformatically correcting for these differences is also complicated as this may change the inflammatory setting of the cells, something undesirable when studying a tissue undergoing inflammatory processes during repair/regeneration [59]. Likewise, the tissue digestion protocol will also enrich certain cell types as well as eliminate others.

Despite the limited sample size of *n* = 3, our scRNA-seq study provides descriptive data on numerous stromal subsets and exploratory routes to investigate their role in endometrial regeneration. Multiple stromal environments exist, which may include different cell types, cell states, ECM compositions and immune settings. Furthermore, our study provides information about the complexity of the perivascular environment, revealing how very few genes are truly unique to one cell population, making it complex to distinguish between progenitor cells, fibroblasts, smooth muscle cells and mural cells. This is particularly the case if gene and protein expression are heavily affected by a microenvironment rather than a specific cell type or function.

## Data Availability

The data presented in this study are available upon request from the corresponding author.

## Supplementary Materials

The following are available online at https://www.mdpi.com/article/10.3390/jpm11060448/s1, Figure S1: Study design, donor distribution and identification of endometrial cell clusters. Figure S2: Selection of cells for the subanalysis of stromal cells and pericytes and expression distribution of THY1. Figure S3: Stromal and perivascular cell populations identified by Vento-Tormo et al.

## Code Availability

Codes used in this study are available at Github at https://carolina-vg.github.io/sics-endo/ (accessed on 19 May 2021).
